# Supplementary material for: Replicative Acinetobacter baumannii strains interfere with phagosomal maturation by modulating the vacuolar pH
Source: bioRxiv. 2023 Feb 2:2023.02.02.526753. Preprint. [Version 1] doi: 10.1101/2023.02.02.526753 (PMC9915592; doi:10.1101/2023.02.02.526753)
Supplement: 1 [file NIHPP2023.02.02.526753V1-supplement-1.pdf]

bioRxiv preprint doi: https://doi.org/10.1101/2023.02.02.526753; this version posted February 2, 2023. The copyright holder for this preprint (which was not certified by peer review) is the author/funder, who has granted bioRxiv a license to display the preprint in perpetuity. It is made available under aCC-BY 4.0 International license.

## Supporting Information

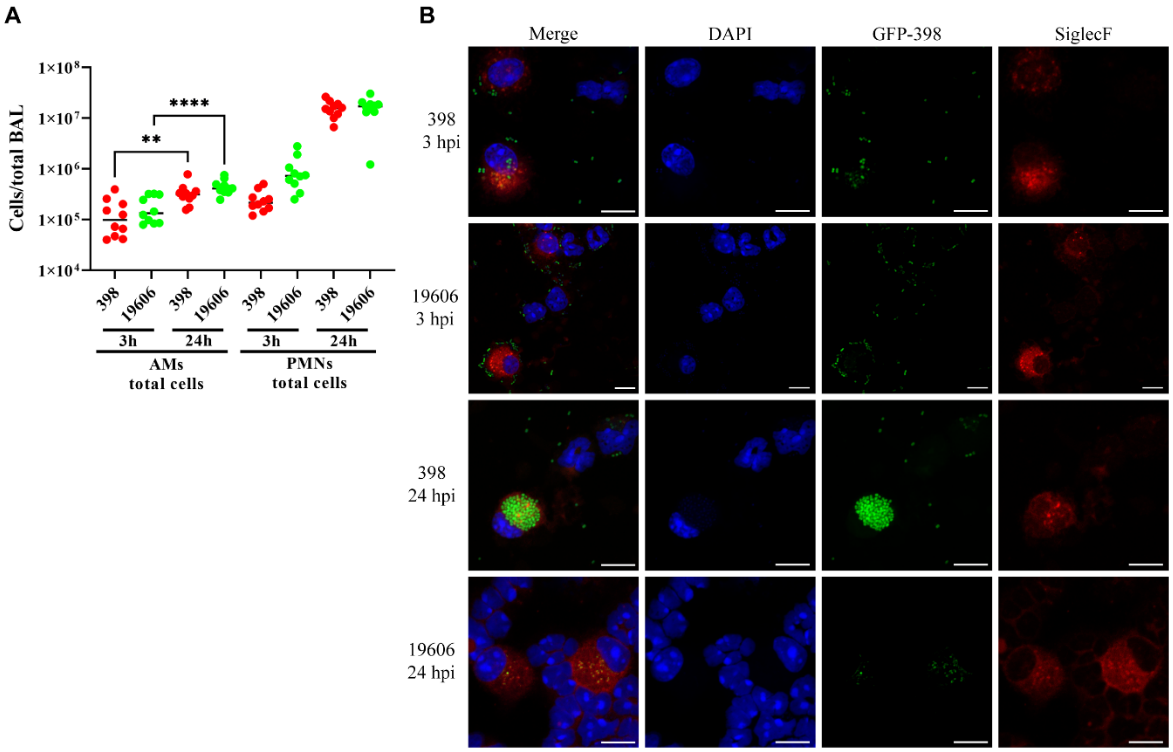


**Figure S1. *A. baumannii* clinical isolate 398 infects AMs *in vivo* and survives inside ACVs.** (A) Quantification of total AMs (CD45+CD11c+SiglecF+CD11b-) and PMNs (CD45+CD11b+Ly6G+) in the BALF of mice infected for 3 h or 24 h with GFP-398 or GFP-19606 strains. (B) Individual channels from confocal micrograph showed in the figure 1D.

bioRxiv preprint doi: https://doi.org/10.1101/2023.02.02.526753; this version posted February 2, 2023. The copyright holder for this preprint (which was not certified by peer review) is the author/funder, who has granted bioRxiv a license to display the preprint in perpetuity. It is made available under aCC-BY 4.0 International license.

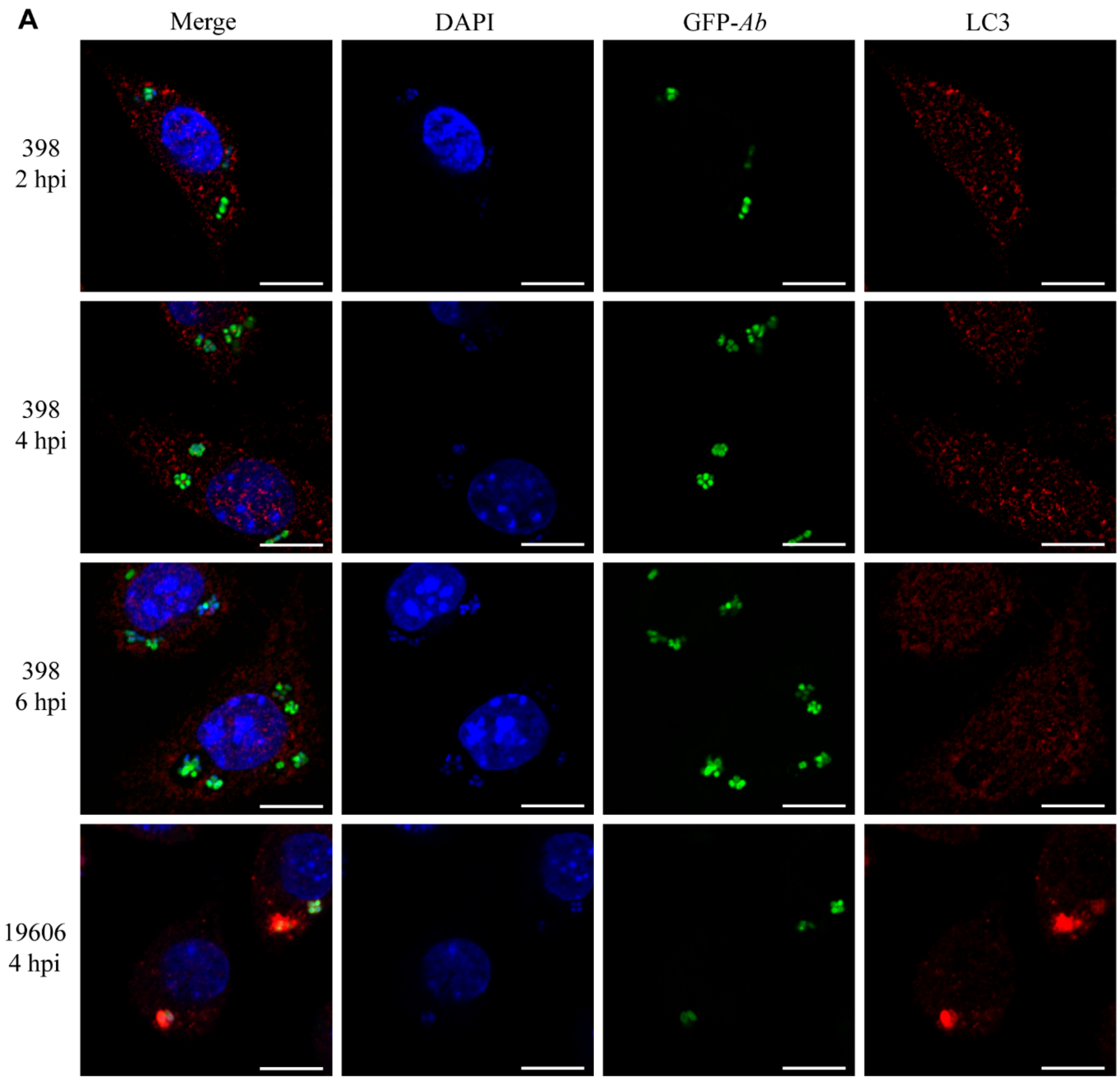


**Figure S2. 398 ACV does not colocalize with the autophagic marker LC3.** (A) Single channel images of the inset micrograph shown in panel 2A. Bars: 10 µm.

bioRxiv preprint doi: https://doi.org/10.1101/2023.02.02.526753; this version posted February 2, 2023. The copyright holder for this preprint (which was not certified by peer review) is the author/funder, who has granted bioRxiv a license to display the preprint in perpetuity. It is made available under aCC-BY 4.0 International license.

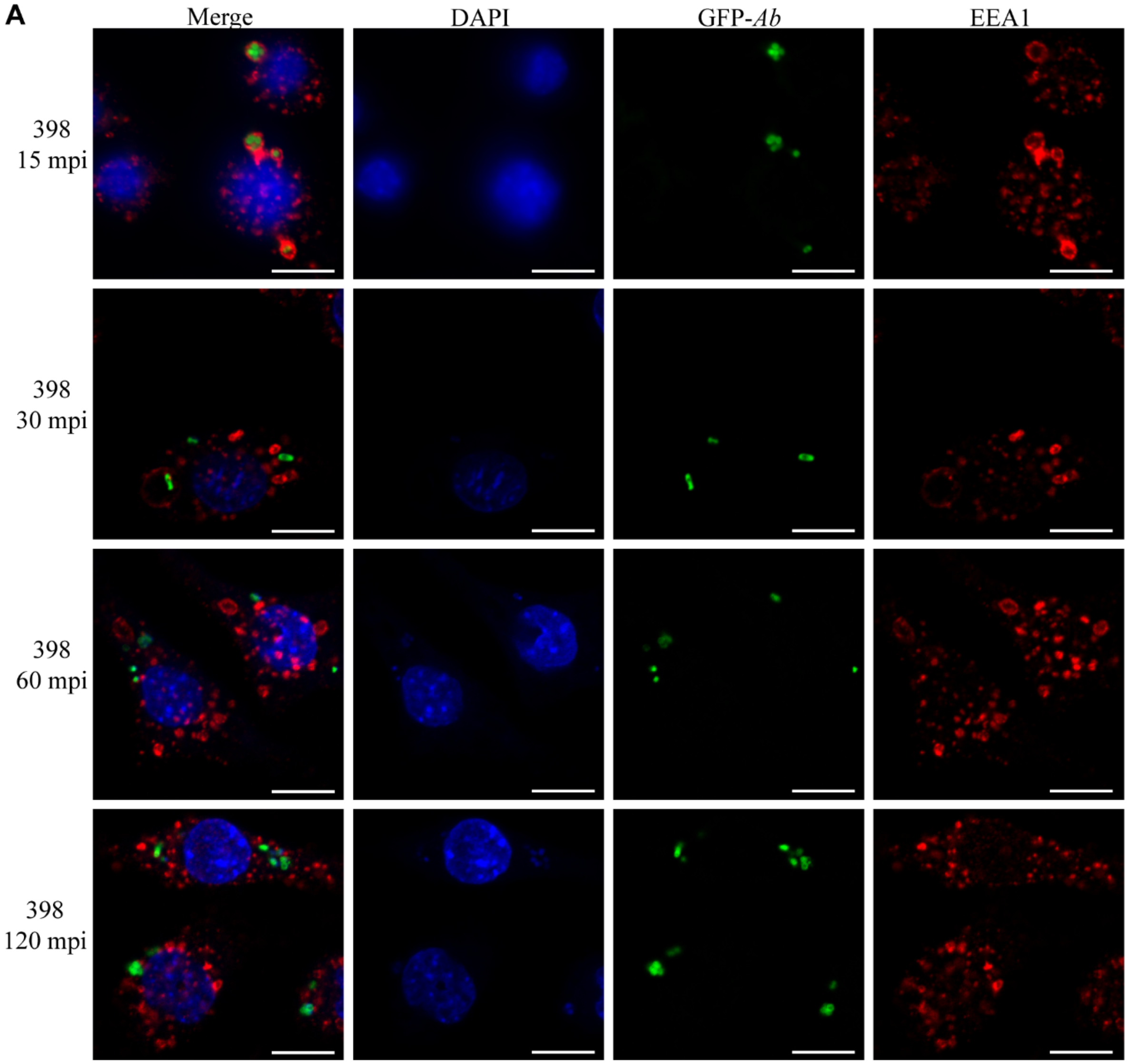


**Figure S3. The ACV interacts with the early marker EEA1.** (A) Single channel images from the inset micrograph shown in figure 3A. Bars: 10 µm.

bioRxiv preprint doi: https://doi.org/10.1101/2023.02.02.526753; this version posted February 2, 2023. The copyright holder for this preprint (which was not certified by peer review) is the author/funder, who has granted bioRxiv a license to display the preprint in perpetuity. It is made available under aCC-BY 4.0 International license.

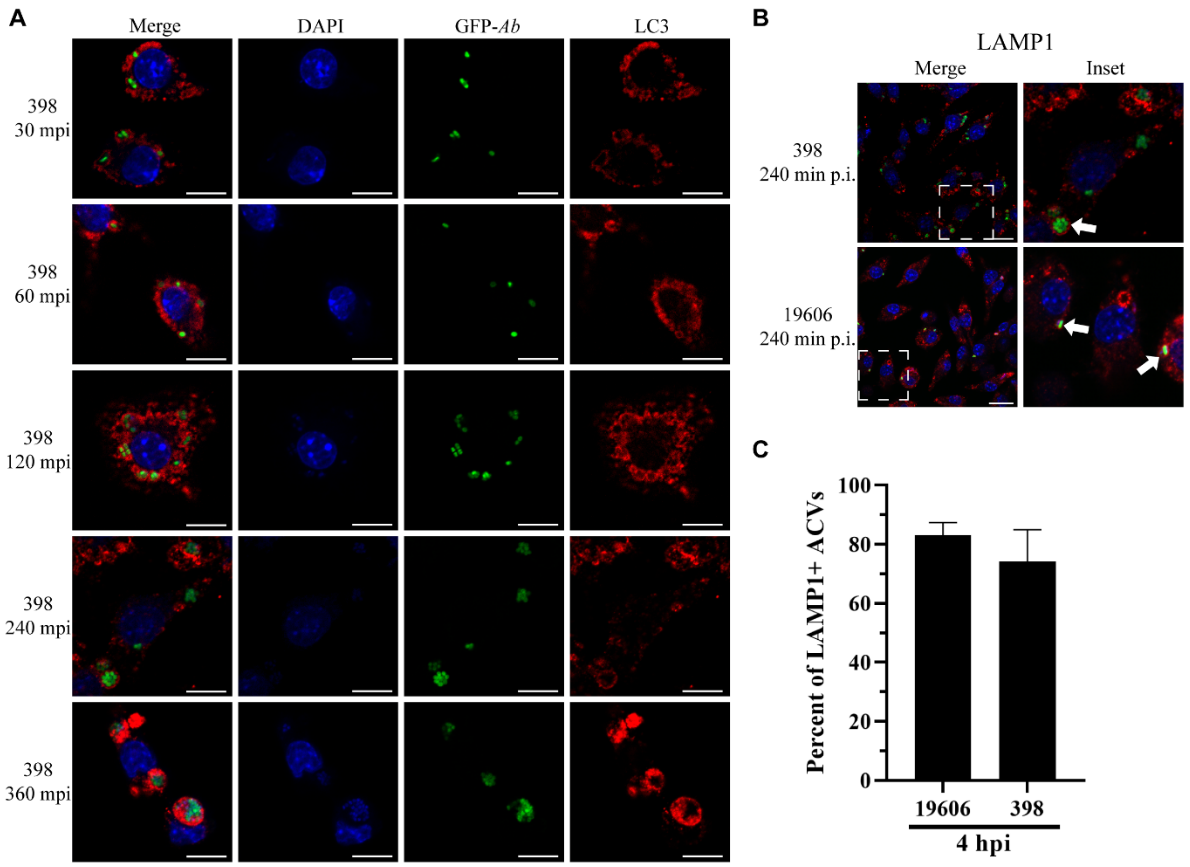


**Figure S4. 398 and 19606 ACVs colocalize with the late marker LAMP1.** (A) Single channel images of the inset micrograph shown in panel 3B. Bars: 10 µm. (B) J774A.1 macrophages were infected with strains GFP-398 or GFP-19606 and fixed 4 hpi. The samples were stained to observe cell nuclei (blue), GFP-*A. baumannii* (green) and LAMP1 (red). Representative confocal images of the infections are shown. White arrows indicate ACVs that colocalize with the marker LAMP1. Bars: 20 µm. Insets (40 µm) are a higher magnification of region indicated in the corresponding image with a white box. (C) Quantification of LAMP1+ 398 or 19606 ACVs. At least 200 infected cells were analyzed. The results are expressed as means ± SEM of three independent experiments.

bioRxiv preprint doi: https://doi.org/10.1101/2023.02.02.526753; this version posted February 2, 2023. The copyright holder for this preprint (which was not certified by peer review) is the author/funder, who has granted bioRxiv a license to display the preprint in perpetuity. It is made available under aCC-BY 4.0 International license.

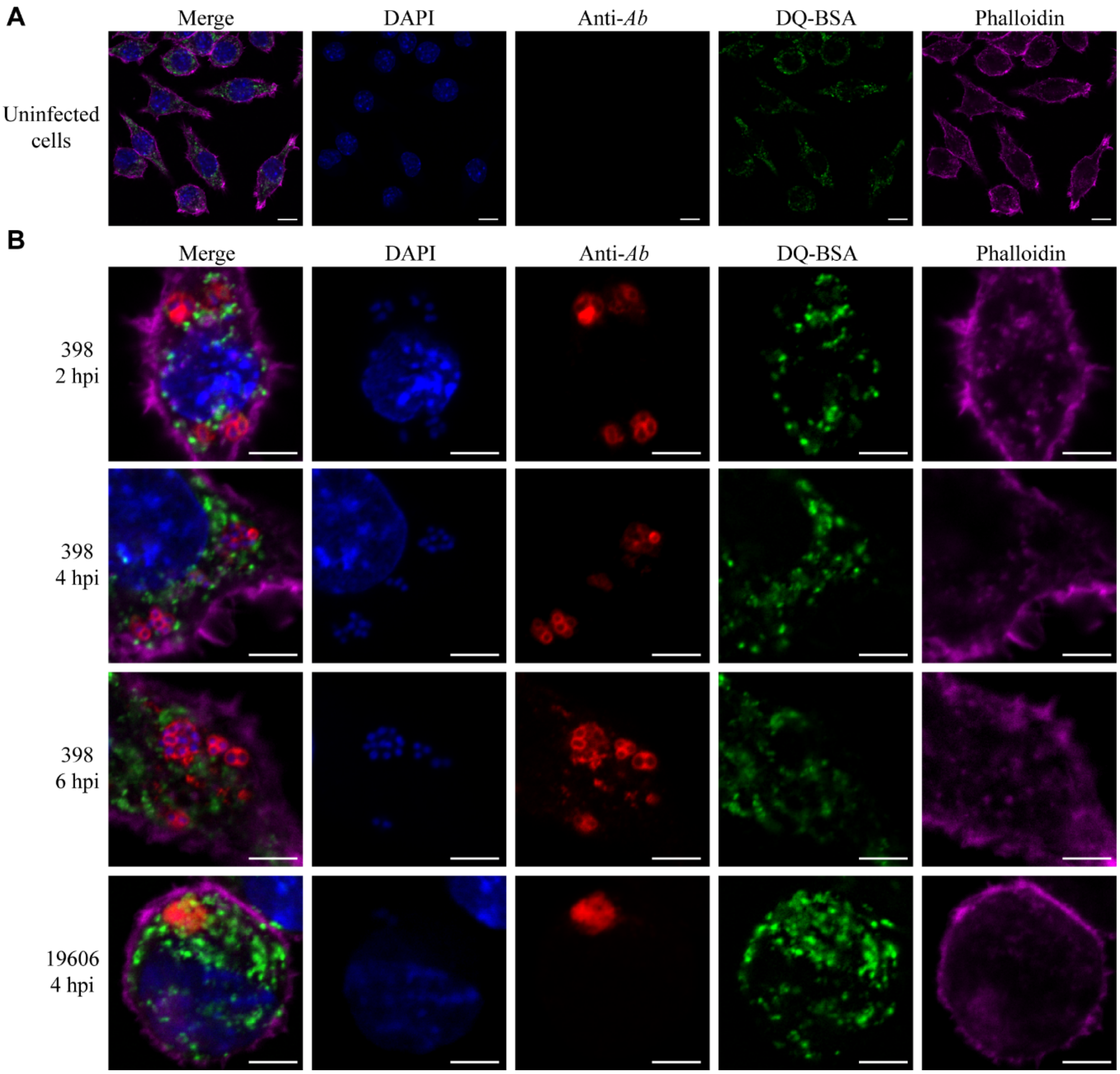


**Figure S5. DQ-BSA green fluorescence in non-infected or *A. baumannii* infected cells.** (A) Representative image of non-infected J774A.1 cells treated with DQ-BSA green. (B) Single channel images of the inset micrograph shown in figure 4A. Bars: 5 µm.

bioRxiv preprint doi: https://doi.org/10.1101/2023.02.02.526753; this version posted February 2, 2023. The copyright holder for this preprint (which was not certified by peer review) is the author/funder, who has granted bioRxiv a license to display the preprint in perpetuity. It is made available under aCC-BY 4.0 International license.

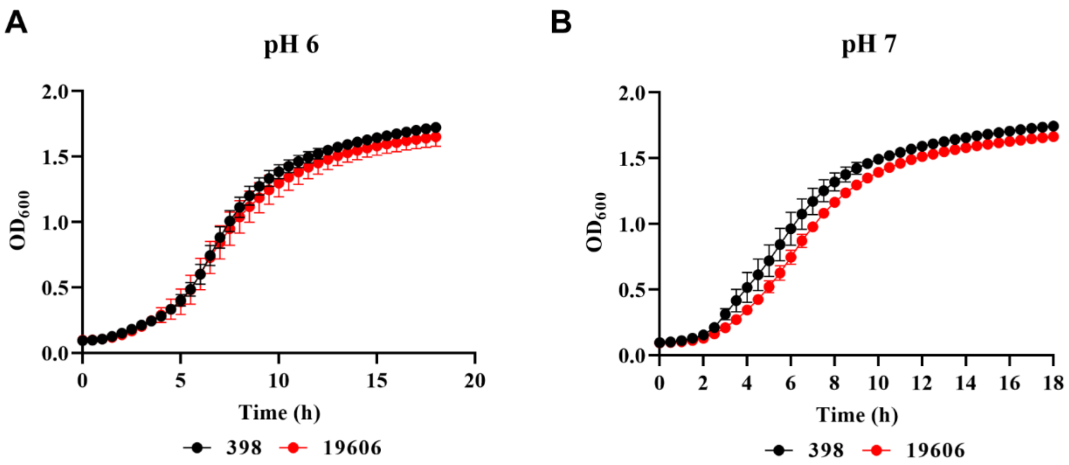


**Figure S6. Growth of *A. baumannii* strains at different pH.** Growth of 398 and 19606 strains in LB buffered at (A) pH 6 or (B) pH 7 was measured by $OD_{600}$.

bioRxiv preprint doi: https://doi.org/10.1101/2023.02.02.526753; this version posted February 2, 2023. The copyright holder for this preprint (which was not certified by peer review) is the author/funder, who has granted bioRxiv a license to display the preprint in perpetuity. It is made available under aCC-BY 4.0 International license.

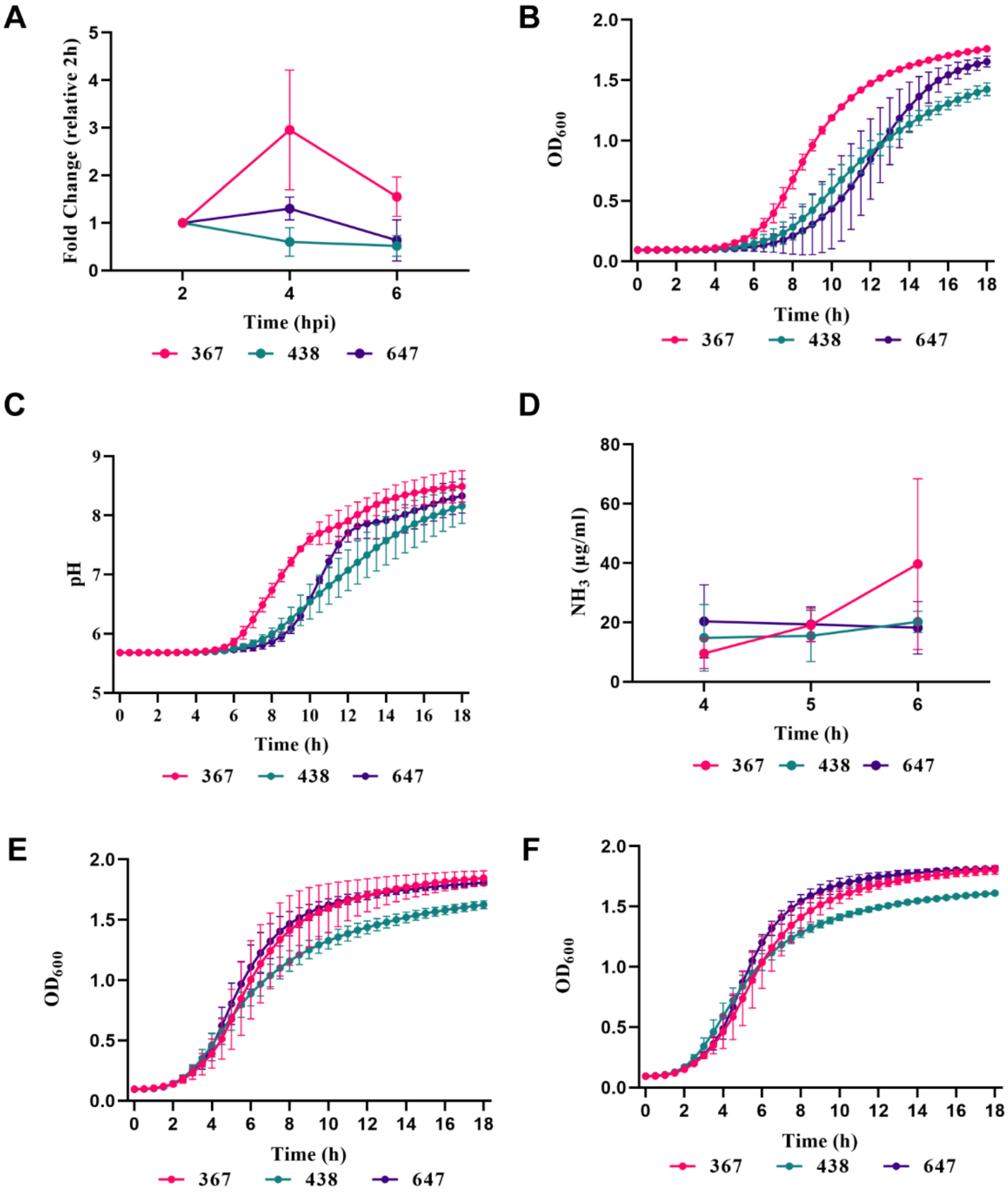


**Figure S7. The replicative capacity of *A. baumannii* clinical isolates is related to growth fitness at acidic pH.** (A) Intracellular replication of *A. baumannii* clinical isolates 367, 438 and 438 in J774A.1 macrophages determined by antibiotic protection assays. Growth of *A. baumannii* strains in LB buffered at (B) pH 5, (E) pH 6 or (F) pH 7 was measured by $OD_{600}$. (C) Changes in culture pH during *A. baumannii* strains growth, determined by phenol red absorbance at 560 nm. (D) Concentration of ammonia in LB cultures of *A. baumannii* strains at 4, 5 and 6 h post-inoculation. Results are expressed as mean ± SEM of three independent experiments.

**Table S1. Bacterial strains and plasmids.**

bioRxiv preprint doi: https://doi.org/10.1101/2023.02.02.526753; this version posted February 2, 2023. The copyright holder for this preprint (which was not certified by peer review) is the author/funder, who has granted bioRxiv a license to display the preprint in perpetuity. It is made available under aCC-BY 4.0 International license.

| Bacterial strain | Description | Reference |
|---|---|---|
| ***Acinetobacter baumannii*** | - | - |
| 19606 | Urinary tract infection isolate | [1] |
| 398 | Urinary tract infection isolate | IHMA Labs. |
| 367 | Urinary tract infection isolate | Enthasis Therapeutics/Allita Miller |
| 438 | Respiratory infection isolate | David Waiss – Emory - Satola, Sarah ssatola@emory.edu |
| 647 | Respiratory infection isolate | David Waiss - Emory - Satola, Sarah ssatola@emory.edu |
| 19606 GFP | GFP-expressing transconjugant | [2] |
| 398 GFP | GFP-expressing transconjugant | [2] |
| 398 mCherry | mCherry-expressing transconjugant | This work. |
| **Plasmid** | - | - |
| pBAV1k-t5-gfp | Published vector, kanamycin resistance marker | [3] |
| pUC18T-mini-Tn7T-Zeo-GFP | promotor promlac and gfp gene from pBAV1k-t5-gfp on pUC18T-mini-Tn7T-Zeo backbone, zeocin resistance marker [2] | [2] |
| pUC18T-mini-Tn7T-Zeo-mCherry | promotor promlac and gfp gene from pBAV1k-t5-mCherry on pUC18T-mini-Tn7T-Zeo backbone, zeocin resistance marker | This work. |

**References**


1. Hugh R, Reese R. Designation of the type strain for Bacterium anitratum Schaub and Hauber 1948. Int J Syst Bacteriol. 1967;17: 245–254. doi:10.1099/00207713-17-3-245.
2. Sycz G, Di Venanzio G, Distel JS, Sartorio MG, Le NH, Scott NE, Beatty WL, Feldman MF. Modern Acinetobacter baumannii clinical isolates replicate inside spacious vacuoles and egress from macrophages. PLoS Pathog. 2021 Aug 9;17(8):e1009802. doi: 10.1371/journal.ppat.1009802.
3. Bryksin A V., Matsumura I. Rational Design of a Plasmid Origin That Replicates Efficiently in Both Gram-Positive and Gram-Negative Bacteria. Mokrousov I, editor. PLoS One. 2010;5: e13244. doi:10.1371/journal.pone.0013244.
